# Supplementary material for: 3D Conformational Generative Models for Biological Structures Using Graph Information-Embedded Relative Coordinates
Source: Molecules. 2022 Dec 31;28(1):321. doi: 10.3390/molecules28010321 (PMC9823299; doi:10.3390/molecules28010321)
Supplement: Supplementary file 1 [file molecules-28-00321-s001.zip › molecules-2055345-supplementary.pdf]

Supporting Information

3D Conformational Generative Models for Biological Structures Using Graph Information-Embedded Relative Coordinates

Mingyuan Xu [1,*,†], Weifeng Huang [2,†], Min Xu [3], Jinping Lei [2,*] and Hongming Chen [1,*]

[1] Guangzhou Laboratory, Guangzhou International Bio Island, Guangzhou 510005, China
[2] School of Pharmaceutical Sciences, Sun Yat-Sen University, Guangzhou 510006, China
[3] XtalPi, International Biomedical Innovation Park II 3F, 2, Shenzhen 518000, China
* Correspondence: mingyuan.xu.sci@gmail.com (M.X.); leijp@mail.sysu.edu.cn (J.L.); chen_hongming@grmh-gdl.cn (H.C.)
† These authors contributed equally to this work.

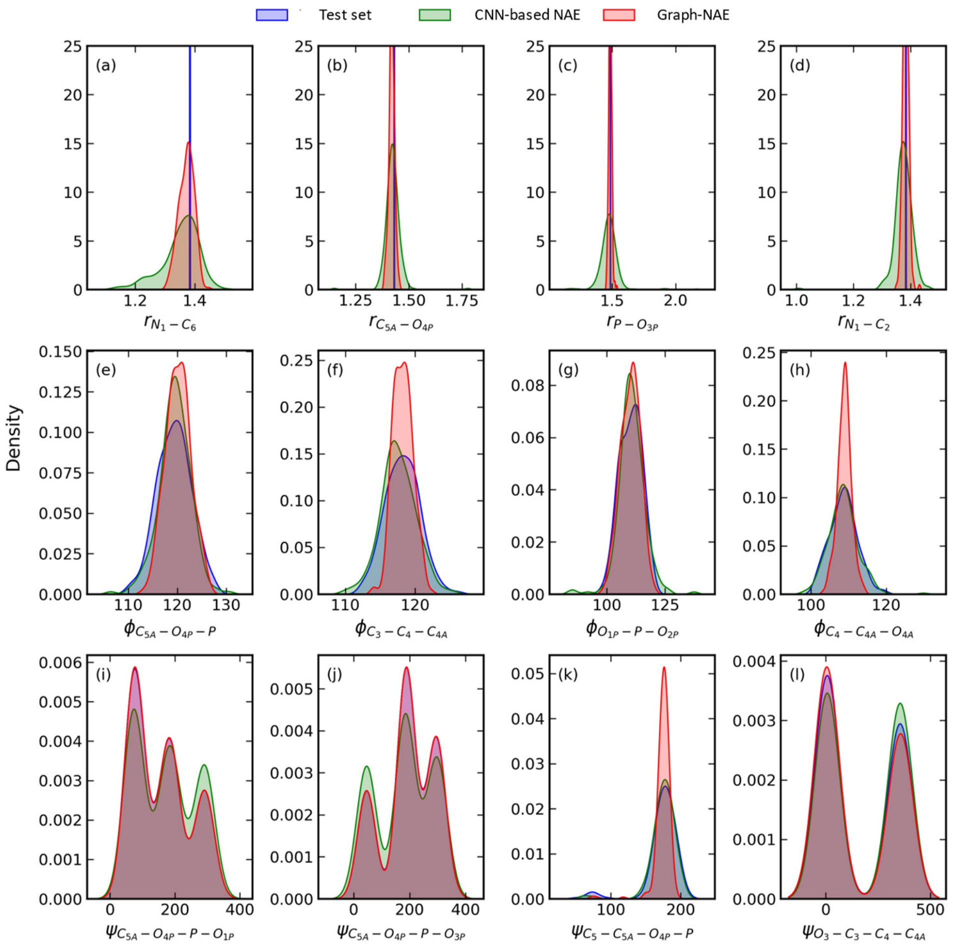

Figure S1. The distribution of 12 randomly selected internal coordinates of PLP in reconstructed conformations of Graph-AE (red) and CNN-based native AE (green) and ground truth of test set (blue).

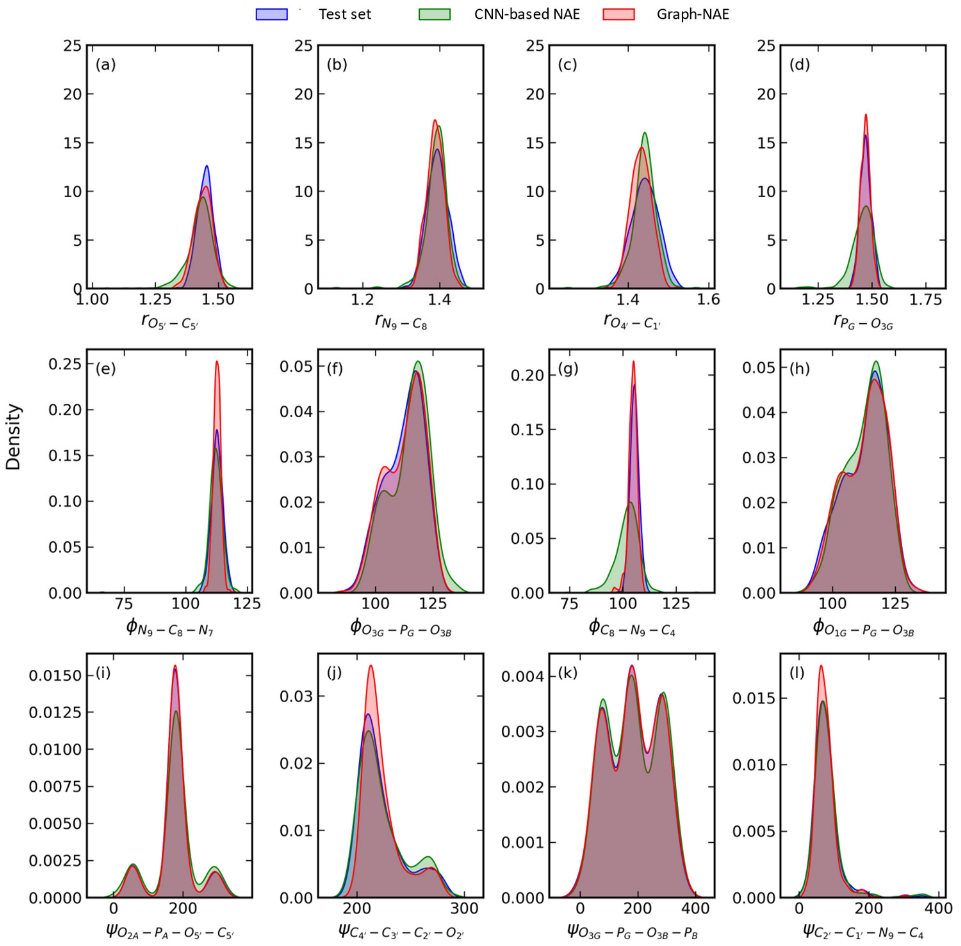

Figure S2. The distribution of 12 randomly selected internal coordinates of ATP in reconstructed conformations of Graph-AE (red) and CNN-based native AE (green) and ground truth of test set (blue).

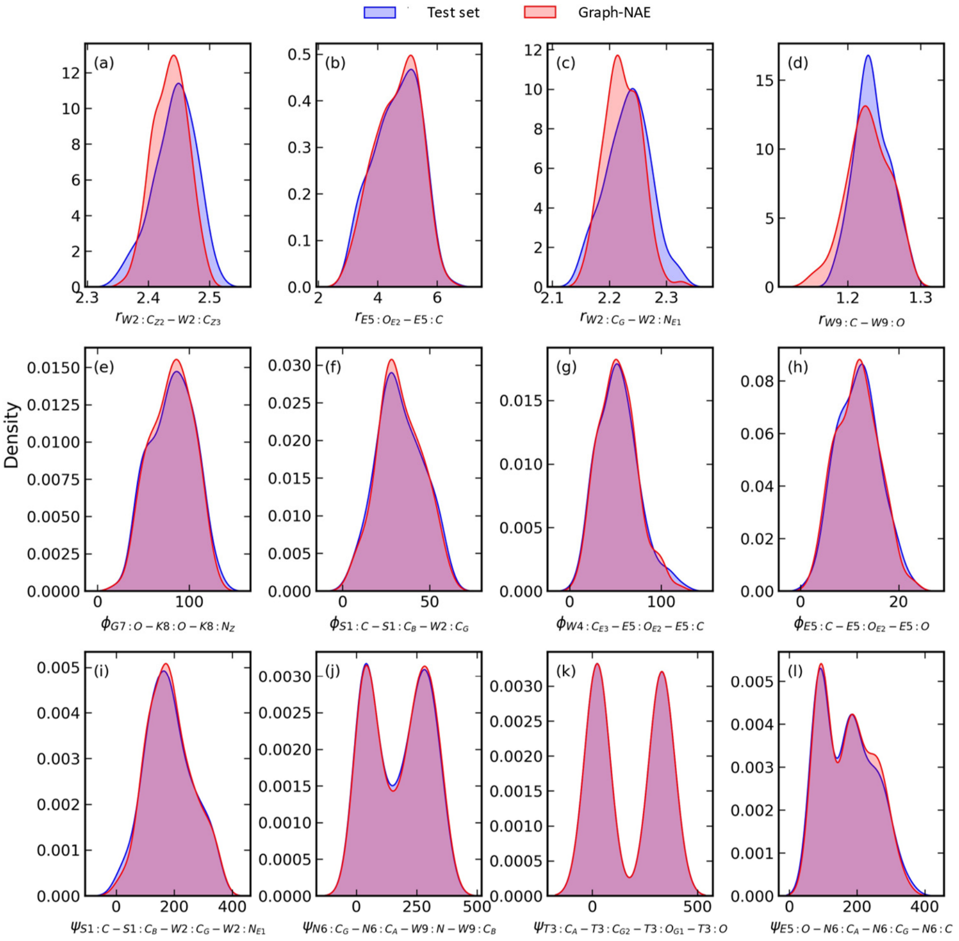

Figure S3. The distribution of 12 randomly selected internal coordinates of 1LE1 in Graph-NAE reconstructions (red) and structures in test set (blue).

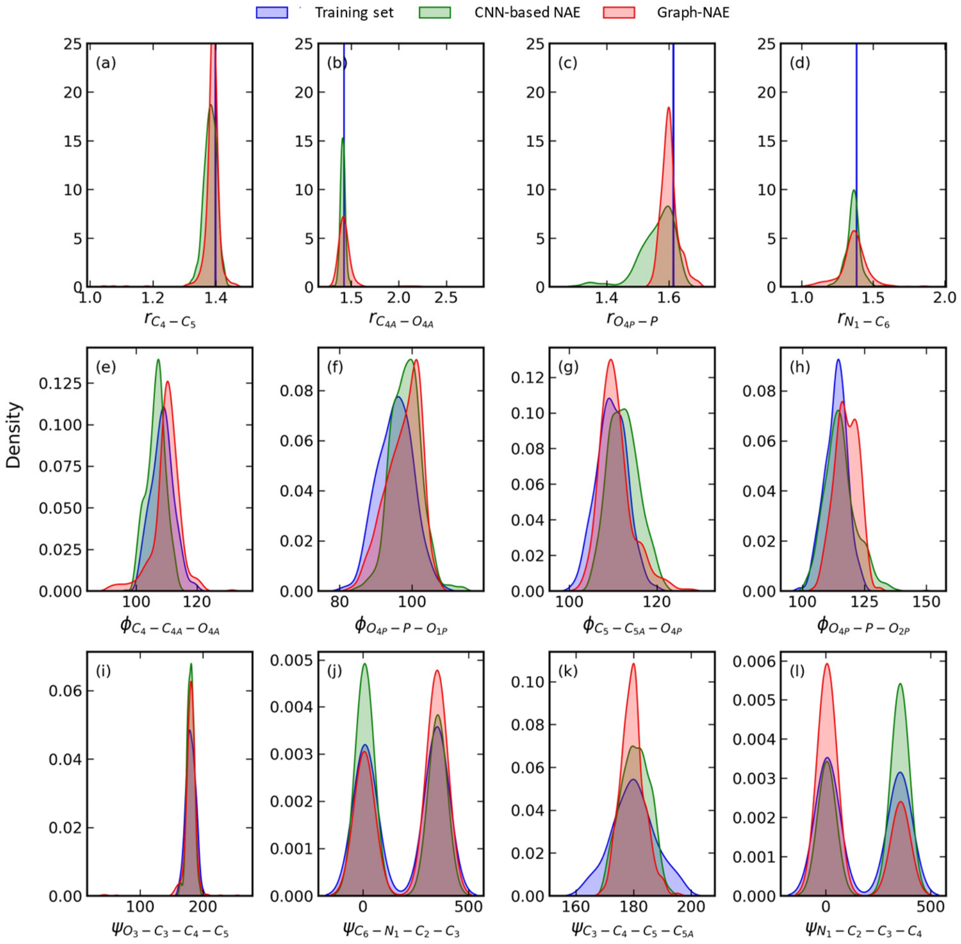

Figure S4. The distribution of 12 randomly selected internal coordinates of PLP in interpolated structure of Graph-AE (red), CNN-based native AE (green) and original structures in training set (blue).

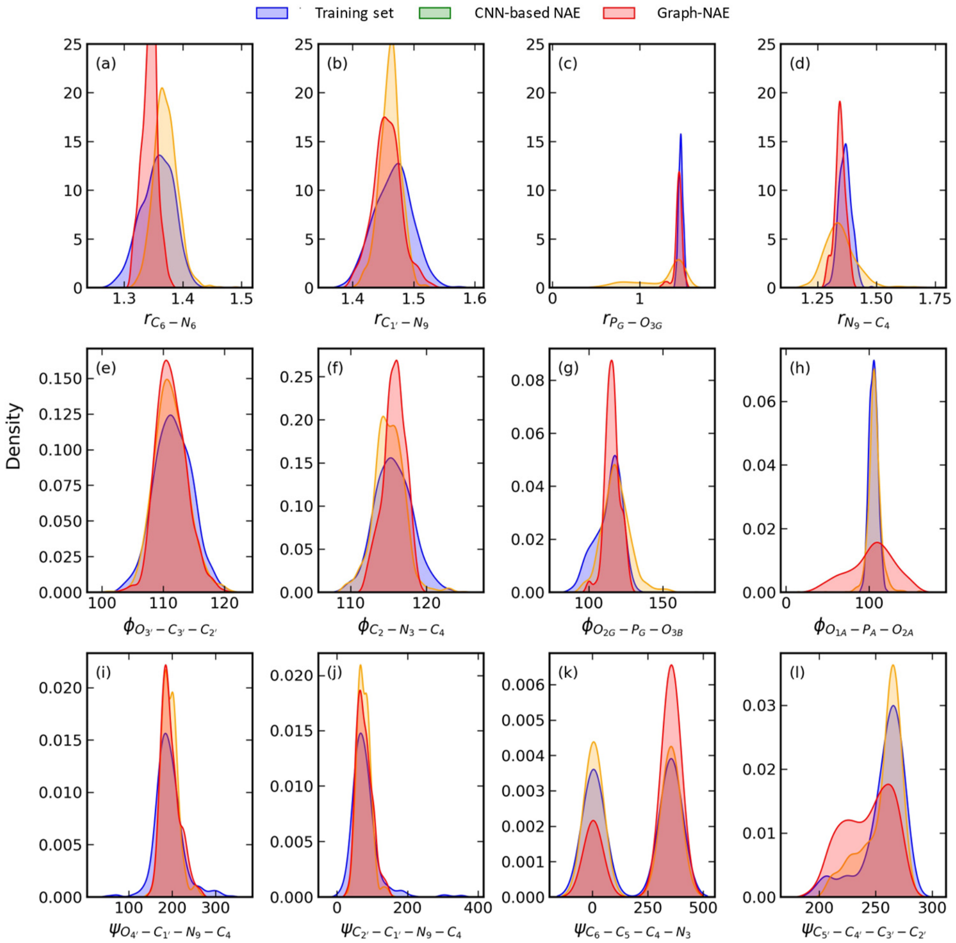

Figure S5. The distribution of 12 randomly selected internal coordinates of ATP in interpolated structure of Graph-AE (red), CNN-based native AE (green) and original structures in training set (blue).

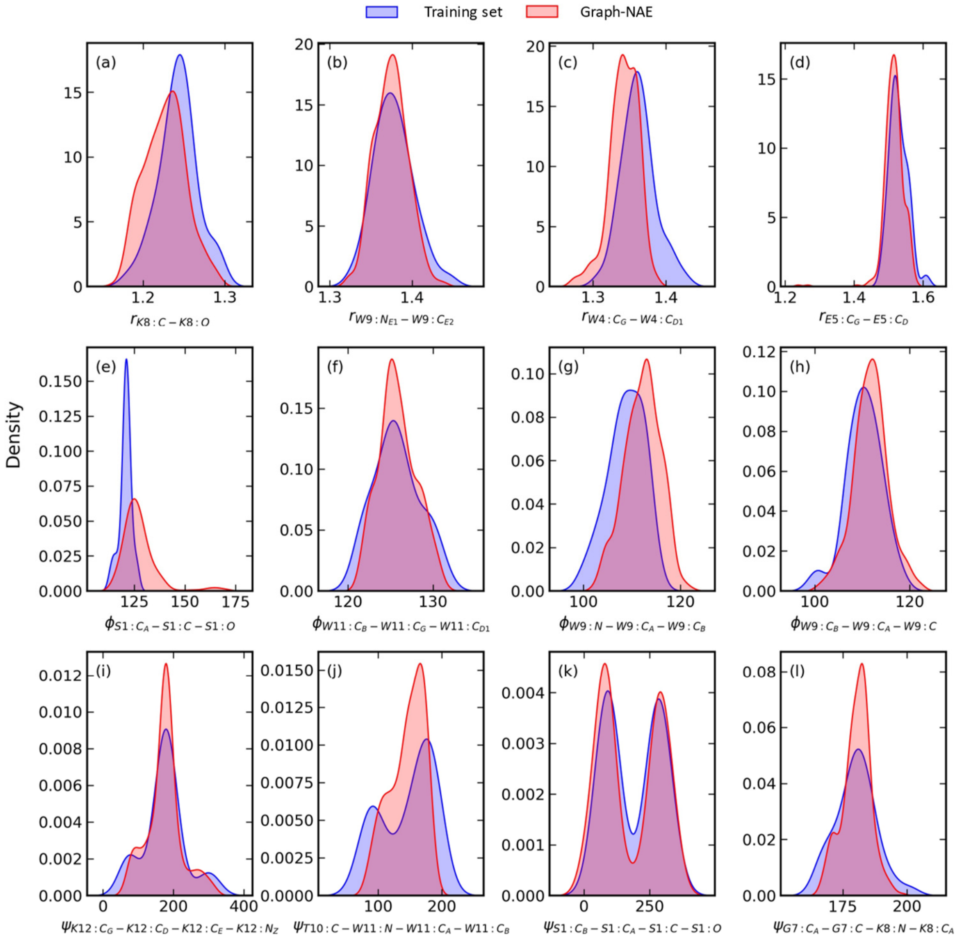

Figure S6. The distribution of 12 randomly selected internal coordinates of 1LE1 in interpolated structure of Graph-AE (red) and original structures in training set (blue).

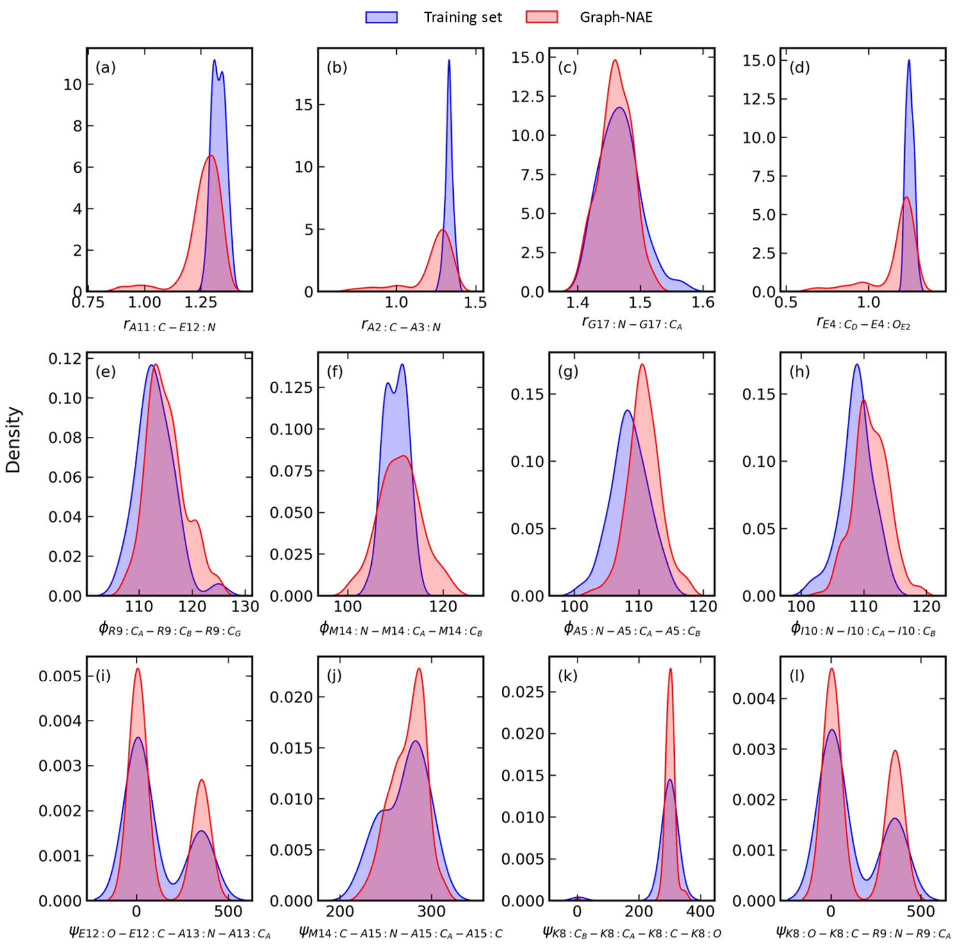

Figure S7. The distribution of 12 randomly selected internal coordinates of 2I9M in Graph-NAE interpolations (red) and structures in training set (blue).
